# Thirty Years of Mentoring by “Bob Chan” of Young Japanese Surgeons to Become Scientists: An Adventure of Love

**DOI:** 10.1002/ags3.70120

**Published:** 2025-11-18

**Authors:** Robert M. Hoffman, Kentaro Miyake, Itaru Endo

**Affiliations:** ^1^ AntiCancer Inc San Diego California USA; ^2^ Department of Surgery University of California San Diego California USA; ^3^ Department of Gastroenterological Surgery Yokohama City University Graduate School of Medicine Yokohama Japan

**Keywords:** A1‐R, fluorescent proteins, friendship, FUCCI, mentorship, PDOX, rMETase, translational oncology

## Abstract

Over the past three decades, we have mentored a generation of young Japanese surgeons, guiding them to become internationally recognized surgeon‐scientists. Through a unique collaboration between Japanese academic institutions and our laboratories at AntiCancer Inc. and the University of California, San Diego (UCSD), these trainees engaged in immersive translational research. They mastered advanced imaging and therapeutic tools including fluorescent‐protein technology, patient‐derived orthotopic xenograft (PDOX) models, the FUCCI cell‐cycle imaging system, tumor‐targeting *Salmonella typhimurium* A1‐R (A1‐R), and recombinant methioninase (rMETase) to treat the methionine addiction of cancer. These technologies enabled real‐time visualization of cancer biology in vivo, accurate modeling of tumor behavior, and the development of strategies to target quiescent, treatment‐resistant cancer cells. FUCCI imaging revealed that most solid cancer cells within tumors exist in non‐cycling states, which are largely unaffected by conventional therapies. Our team explored methods to decoy these cells into vulnerable phases of the cell cycle. PDOX models provided a clinically‐relevant platform to evaluate such strategies, while A1‐R and rMETase offered novel therapeutic avenues by exploiting cancer‐specific vulnerabilities. The present review highlights the scientific advances achieved through this collaboration, but also the human story of mentorship, cultural exchange, and the formation of a lasting international academic network and permanent friendship. Many of our mentees now lead research laboratories and academic departments across Japan, continuing the cycle of innovation and global partnership. We reflect on this journey as a successful model for training surgeon‐scientists and advancing precision cancer therapy through visualization, imagination, and mentorship.

## Abstract 2

This article reflects on a 30‐year collaboration between Bob Chan's laboratory in San Diego and Japanese surgical institutions, highlighting the mentorship of young surgeons who became surgeon‐scientists. Through the development of advanced models such as PDOX models, FUCCI cell‐cycle imaging, A1‐R bacteria, and rMETase, this partnership fostered both scientific innovation and the academic surgeon mindset. The narrative underscores the value of international exchange in revitalizing surgical research and inspiring future generations.

## 1 Introduction

At the time of the beginning of our collaboration more than 30 years ago in Japan, academic surgery often followed rigid hierarchies, with few opportunities for international training. The culture was focused on mastering operative techniques, and less on integrating research into surgical careers. For many of our Japanese student‐surgeons, arriving at our San Diego laboratory was their first experience abroad and marked a turning point in their professional and personal lives. We cultivated a unique environment that emphasized creativity, freedom of thought, and visual scientific communication. This open, collaborative spirit gave rise to lifelong cross‐cultural mentorship, friendship and research partnerships that continue today. In addition to laboratory research, many trainees presented their work at international conferences, co‐authored high‐impact publications, and brought these skills back to Japan—changing the academic surgical landscape from within.

In the early 1990s, a unique bridge was built between Japan and the United States—not by government, but by science. One of us (Robert M Hoffman), affectionately known by Japanese colleagues as “Bob Chan,” began inviting young Japanese surgeons to his Laboratory at AntiCancer Inc. in San Diego. There, these surgeons were not only trained in the fundamentals of cancer biology, but also introduced to cutting‐edge imaging and translational research techniques.

At the time, the concept of “an academic surgeon” was still emerging in Japan. Surgical training was rigorous and deeply hierarchical, with limited opportunities for academic exploration abroad. Our mentorship offered something rare: the freedom to question, to experiment, and to innovate. The impact of our guidance extended far beyond technical training. Many of these trainees went on to lead laboratories and departments, develop original cancer models, and influence surgical education in Japan. This 30‐year journey represents a remarkable chapter in international academic collaboration and scientific mentorship.

This review highlights the key scientific tools and discoveries that emerged from this partnership. These include the development and application of fluorescent proteins in live imaging, the establishment of patient‐derived orthotopic xenograft (PDOX) mouse models of cancer, real‐time cell cycle visualization with FUCCI, and the novel use of *Salmonella typhimurium* A1‐R (A1‐R) and recombinant methioninase (rMETase) for targeted cancer therapy.

**FIGURE 1:**
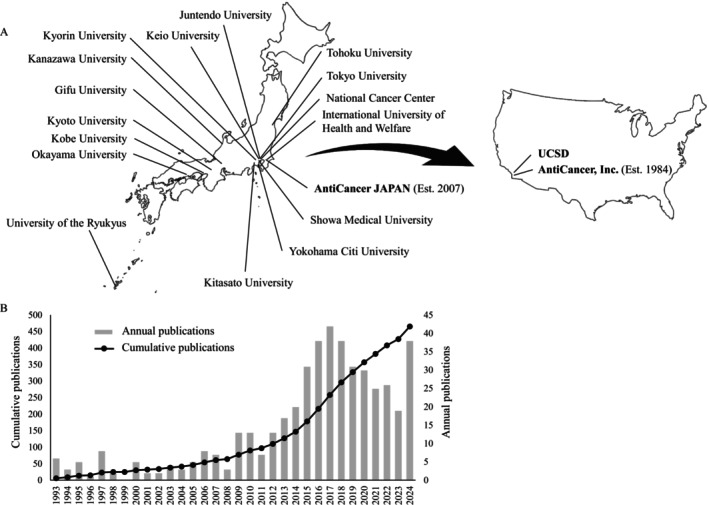
Expansion of collaborations between Bob Chan's laboratory and Japanese institutions. (A) Geographical distribution of major collaborating universities in Japan, connected to AntiCancer Inc. (San Diego, USA) by an arrow, illustrating the flow of surgeon‐scientists since 1994. (B) Annual (bars) and cumulative (line) number of publications by Japanese surgeons trained in the laboratory from 1993 to 2024.

Over the past three decades, the collaboration between Bob Chan's laboratory at AntiCancer Inc. and Japanese surgical institutions has expanded both geographically and academically. This partnership began with the arrival of the first Japanese surgeon in 1994 and has since involved multiple universities across Japan, resulting in numerous joint publications and technological innovations. The geographical scope and chronological growth of these collaborations are summarized in Figure 1.

## 2 Fluorescent‐ Protein Imaging In Vivo

**FIGURE 2:**
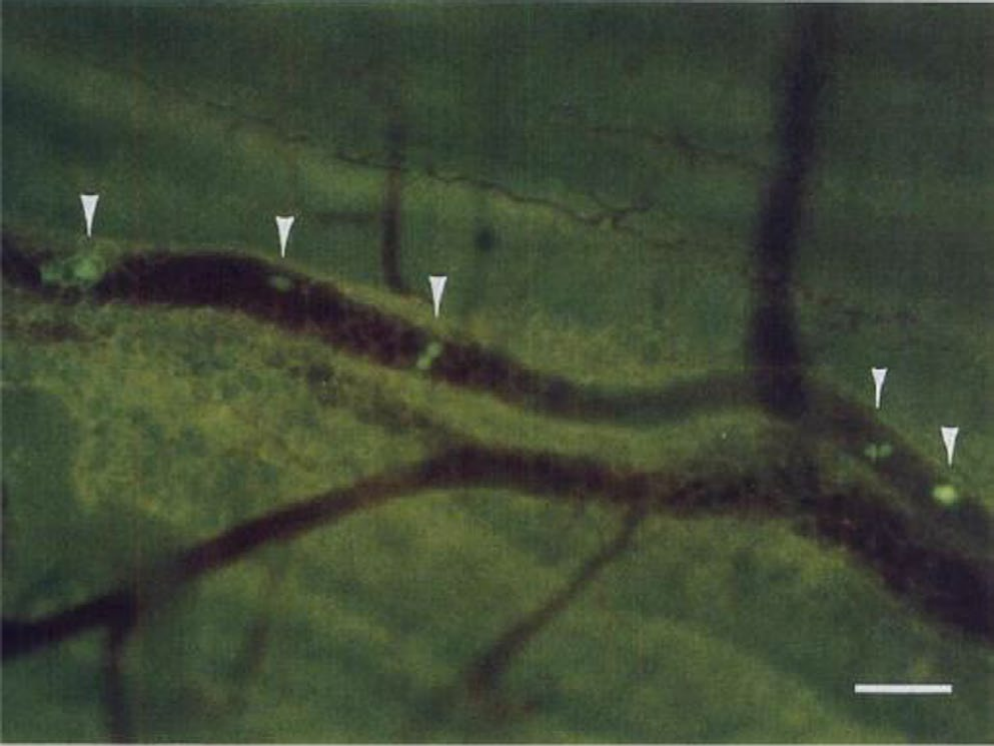
GFP‐expressing cancer cells in veins and capillaries. To study the limit of detection of GFP‐expressing cancer cells in vivo, a nude mouse was sacrificed 2 min after tail vein injection of Colon‐38 mouse colon cancer cells. The fresh organ tissues removed from the mice were directly observed under fluorescence microscopy with no treatment. Scale bar: 100 μm. Arrowheads: GFP‐expressing Clone‐38 cells in a peritoneal vessel. Reproduced from Chishima et al. [1].

Our invention of the use of fluorescent proteins in vivo extended beyond Green fluorescent protein (GFP). Red fluorescent protein (RFP), cyan fluorescent protein (CFP), and yellow fluorescent protein (YFP) variants enabled multiplex imaging—visualizing interactions between and within cancer cells, including nuclear‐cytoplasm interaction within cancer cells with nuclei labeled with our colored fluorescent protein and the cytoplasm labeled with one color fluorescent protein, with immune cells, and with stromal elements in real time (Figure 2) [1]. In hepatobiliary‐pancreatic cancers, these tools allowed us to trace metastatic spread to the liver, lymphatics, and peritoneum with single‐cell and subcellular resolution in vivo [2, 3, 4]. We also developed dual‐color systems to distinguish cancer cell subpopulations within the same tumor, revealing their differential responses to treatment. Such studies led to a deeper understanding of tumor heterogeneity and evolution under therapeutic pressure. The introduction of fluorescent proteins into in vivo cancer research revolutionized the visualization of tumor biology in real time. GFP, originally isolated from the jellyfish *Aequorea victoria* , was first applied in living animal models by our laboratory in the mid‐1990s [1]. By genetically labeling cancer cells with GFP or its color variants—red, cyan, yellow—researchers were able to noninvasively track tumor growth, angiogenesis, invasion, and metastasis in live mice [1, 5, 6, 7, 8, 9].

The significance of this approach lies in its ability to move beyond static histopathology and into the dynamic, spatiotemporal understanding of tumor behavior. For example, fluorescence imaging enabled high‐resolution, intravital microscopy to visualize cancer cell–stromal interactions, drug response, and even single‐cell migration in vivo [5, 10, 11, 12, 13, 14, 15]. These techniques allowed young Japanese surgeon‐scientists to explore cancer as a living, evolving system—providing insight that reshaped their perspective on both basic science and clinical decision‐making.

Furthermore, multicolor fluorescent proteins facilitated the simultaneous imaging of multiple cell types within the tumor microenvironment [15, 16, 17]. This enabled a systems‐level understanding of cancer progression and treatment response, laying the groundwork for later developments such as the FUCCI cell‐cycle‐phase imaging system and imageable PDOX models [16]. In this way, fluorescent protein imaging became not only a tool but a philosophy: to see cancer in action, and to act accordingly.

## 3 Patient‐Derived Orthotopic Xenograft (PDOX) Mouse Model of Cancer

PDOX models were not only anatomically faithful, but also critical in preclinical drug screening [18, 19]. We tested combinations of standard chemotherapy with targeted agents, A1‐R, and rMETase on gastrointestinal cancer and sarcoma models [20, 21, 22, 23, 24, 25]. In many cases, the PDOX response closely mirrored that of the patient, supporting its use in personalized medicine. Additionally, we trained Japanese surgeons in the microsurgical techniques required for PDOX creation, empowering them to establish similar models upon returning home. This led to the rapid spread of PDOX in Japanese academic centers and the development of PDOX‐derived organoids and 3D co‐culture systems.

**FIGURE 3:**
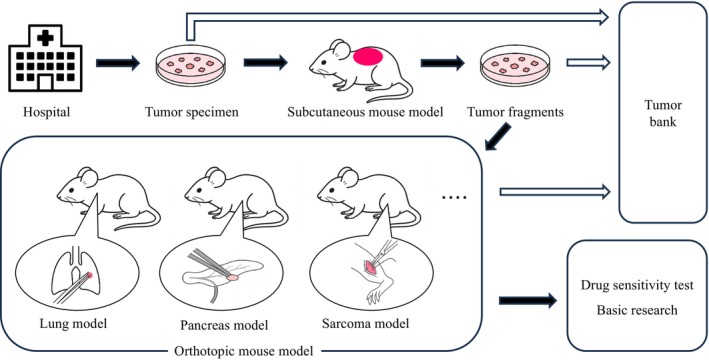
Workflow for the establishment of patient‐derived orthotopic xenograft (PDOX) mouse models. Surgical tumor specimens obtained in the hospital are transported and processed into tumor fragments. Tumors are preserved in the tumor bank and/or used to create a subcutaneous‐tumor mouse model. Fragments from the subcutaneous tumors are subsequently implanted orthotopically into target organs, such as lung, pancreas, or skeletal muscle (sarcoma model), to establish PDOX models. These PDOX models are utilized for drug sensitivity testing and basic research to develop individualized and novel cancer therapies.

To clarify the PDOX creation process, the stepwise procedure for establishing a PDOX model is illustrated in Figure 3. Briefly, surgical specimens obtained from patients are transported under sterile conditions from the hospital to the laboratory, where they are dissected into small tumor fragments. These fragments are either cryopreserved in a tumor bank or implanted subcutaneously into immunodeficient mice to expand the tumor tissue. Tumors from the subcutaneous models are subsequently orthotopically implanted into the anatomically corresponding organ (e.g., stomach, pancreas, liver, lung, skeletal muscle) using surgical orthotopic implantation (SOI) techniques. The resulting PDOX models are then utilized for preclinical drug‐sensitivity testing and mechanistic basic research.

The PDOX model represents a major advance in cancer modeling and precision therapy. Developed and refined in our laboratory, PDOX involves the surgical orthotopic implantation (SOI) of tumor fragments derived from patient specimens into anatomically‐corresponding sites in immunodeficient mice [19]. This methodology faithfully recapitulates the clinical behavior of human cancers, including their metastatic patterns and therapeutic responses.

In contrast to conventional subcutaneous tumor xenografts, PDOX tumors grow within the native tissue microenvironment, preserving interactions between cancer cells, stroma, vasculature, and host immune elements. This orthotopic context is essential for accurately modeling tumor invasion, angiogenesis, and drug resistance—factors often lost in ectopic models [19].

Through hands‐on experience with PDOX, Japanese surgeon‐scientists gained both technical expertise in microsurgical tumor implantation and a deeper understanding of translational oncology. Many applied PDOX to their research upon returning to Japan, leading to a wave of clinically‐relevant preclinical studies in pancreatic, gastric, sarcomatous, and colorectal cancers [22, 23, 26, 27, 28, 29]. These efforts facilitated the evaluation of novel therapeutic strategies, including combination therapies with A1‐R bacteria, rMETase, and chemotherapeutics [23, 30].

PDOX models not only provided a functional model for individual patient tumors but also strengthened the conceptual bridge between bench and bedside.

## 4 FUCCI (Fluorescence Ubiquitination Cell Cycle Indicator) Cell‐Cycle‐Phase Imaging

FUCCI (Fluorescence Ubiquitination Cell Cycle Indicator) utilizes cell cycle‐dependent fluorescent reporters—typically red for G1/G0 phase and green for S/G2/M phase—to visualize the cell‐cycle progression of individual living cancer cells in real time. Despite decades of chemotherapeutic innovation, one of the major challenges in oncology remains the persistence of quiescent, chemotherapy‐resistant cancer cells within tumors. To address this, our team incorporated the FUCCI system—a powerful imaging technology developed by Miyawaki et al.—into in vivo tumor models, including PDOX. We further explored cell‐cycle imaging systems, which provided a precise distinction of G0, G1, S, and M phases [31]. When applied to orthotopic tumor models, FUCCI revealed a striking and clinically‐significant finding: the majority of cells within solid tumors are not actively proliferating, residing instead in a quiescent state that renders them highly resistant to conventional cytotoxic therapies. In three‐dimensional culture and in vivo models, FUCCI imaging revealed that a vast majority of tumor cells existed in G0/G1 arrest, especially after chemotherapy [32]. To overcome this, we employed cell‐cycle decoy strategies using A1‐R to decoy cells into S phase, where cytotoxic agents were most effective [33, 34, 35]. These experiments guided clinical interest in sequential or combination therapies based on cell‐cycle imaging—a concept that is currently being explored for clinical translation. Although no formal clinical trials have yet been initiated, translational feasibility studies have demonstrated that FUCCI can noninvasively monitor tumor cell‐cycle dynamics and correlate with proliferation imaging modalities such as [^18^F]‐FLT PET/CT [36, 37]. These findings support its potential future application for intraoperative visualization of tumor margins and residual cancer cells in solid tumors.

**FIGURE 4:**
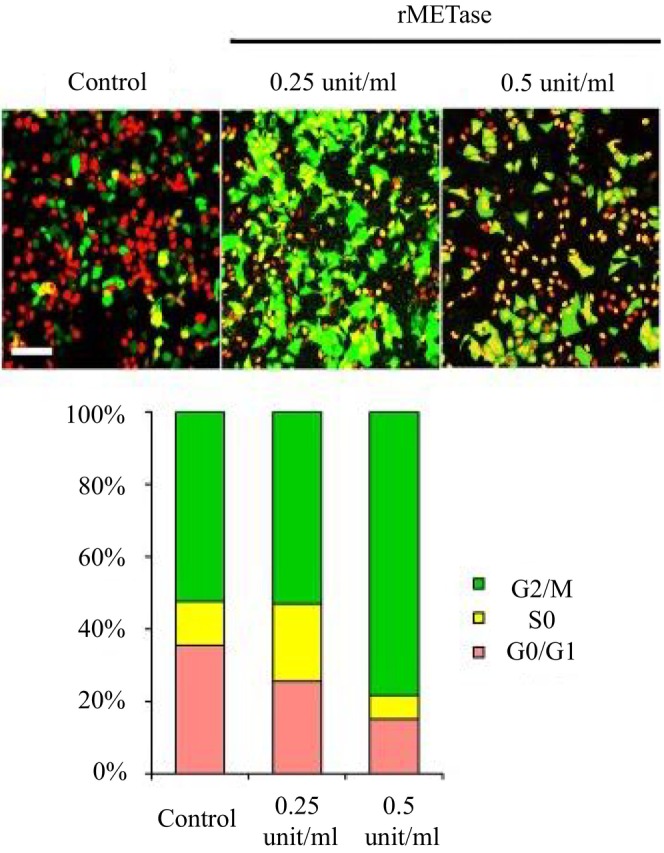
rMETase traps cancer cells in S/G2 phase. After seeding on 35 mm glass dishes and culture over night, MCF‐7 cells were treated with rMETase, at the indicated doses, for 48 h. Histogram shows the percentages of cells in G_1_ phase (red), early‐S phase (yellow), or late‐S/G_2_/M phase (green). Cells at each cell cycle phase were quantitatively assessed by counting the number of cells with each color. *N* = 5 experiments were analyzed. Scale bars: 50 μm. Reproduced from Yano et al. [16].

This insight led to a paradigm shift. Rather than targeting only proliferative cells, strategies were developed to “decoy” quiescent cells into the cell cycle, rendering them susceptible to treatment (Figure 4) [16]. FUCCI enabled researchers to test such approaches dynamically and visually—monitoring responses to decoy drugs, timing of cell‐cycle transition, and the effectiveness of synchronized chemotherapy to target decoyed cancer cells [38].

For Japanese trainees, FUCCI provided a vivid demonstration of how live imaging could guide therapeutic innovation. Several mentees subsequently adapted FUCCI in Japan to study gastric, liver, and colorectal cancer models, deepening their understanding of tumor heterogeneity and resistance mechanisms [37].

FUCCI thus exemplifies how visualization technologies can drive conceptual breakthroughs, bridging basic cell biology with clinical relevance.

## 5 Tumor‐Targeting A1‐R

**FIGURE 5:**
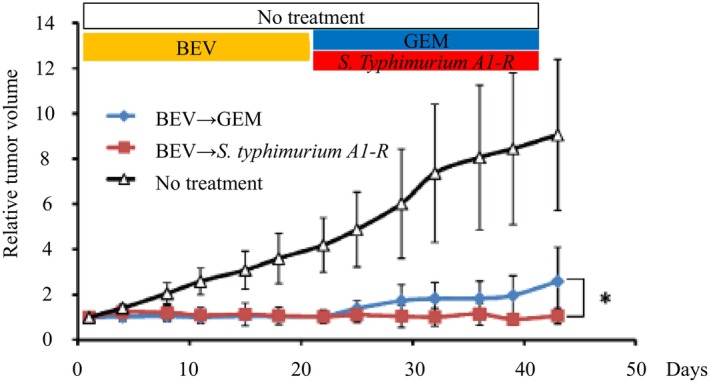
Bevacizumab (BEV) combined with *S. typhimurium* A1‐R arrests a pancreatic cancer PDOX mouse model. BEV significantly reduced the growth of the MiaPaCa‐2 tumor compared to the control on Day 22 (*p* < 0.001). BEV followed by gemcitabine (GEM), and BEV followed by S. *typhimurium* A1‐R significantly reduced MiaPaCa‐2 tumor growth compared to the control group on Day 43 (*p* = 0.001). BEV followed by *S. typhimurium* A1‐R significantly reduced the MiaPaCa‐2 tumor growth compared to BEV followed by GEM (*p* = 0.037). Hiroshima et al. [43].

A1‐R is an attenuated, tumor‐targeting bacterial strain engineered by our group to selectively target and proliferate in tumor tissue while sparing normal organs [39]. A1‐R, auxotrophic for leucine and arginine, was originally derived from *Salmonella typhimurium* and further developed to act as a biological Trojan horse within the tumor microenvironment. When injected systemically into tumor‐bearing mice, A1‐R targets and replicates within solid tumors due to their hypoxic and immunosuppressive milieu, leading to direct cytotoxic effects through tumor lysis. In addition to its direct tumor lysis, A1‐R modulated the tumor immune microenvironment [40]. We observed increased infiltration of innate immune cells following A1‐R colonization, suggesting immunogenic modulation. Moreover, A1‐R combined with chemotherapy or methionine depletion often led to complete tumor regression in PDOX models [21, 23, 40, 41, 42] (Figure 5). We recently engineered A1‐R to deliver recombinant methioninase to the tumor microenvironment [44]. We envision future A1‐R clinical use in combination immunotherapy regimens, especially for refractory cancers such as triple‐negative breast cancer, pancreatic cancer, and sarcomas.

For Japanese surgeon‐scientists, A1‐R provided a fascinating avenue to explore microbial‐based cancer therapy. The ability to visualize GFP‐expressing A1‐R within fluorescent tumors engineered with RFP further underscored the power of combining color‐coded imaging with therapeutic strategies [45]. Trainees gained experience not only in bacterial handling and systemic delivery, but also in evaluating complex host–microbe–tumor interactions using real‐time imaging.

A1‐R therapy is emblematic of our broader vision: to go beyond conventional boundaries and embrace interdisciplinary, sometimes radical, approaches to cancer treatment. The clinical potential of A1‐R remains under active investigation, and several mentees continue to pursue its application in Japan.

## 6 Recombinant Methioninase (rMETase)

Methionine addiction is a fundamental and near‐universal metabolic defect in cancer cells, first extensively characterized by us [46]. Unlike normal cells, which can grow under methionine‐restricted conditions, cancer cells require high levels of methionine for proliferation, particularly due to aberrant transmethylation activity, especially DNA/histone methylation [47, 48]. This phenomenon of methionine addiction in cancer has been termed the “Hoffman effect,” analogous to the Warburg effect of glucose metabolism [49, 50].

To exploit this vulnerability, our lab developed recombinant methioninase (rMETase), an enzyme derived from *Pseudomonas putida* that degrades methionine into α‐ketobutyrate, methanethiol, and ammonia [51, 52]. When administered systemically or orally, rMETase reduces plasma and intra‐tumoral methionine levels [53, 54], selectively arresting cancer cells in late S/G2 phase [16]. This state sensitizes tumors to cytotoxic chemotherapy and other targeted agents [55].

**FIGURE 6:**
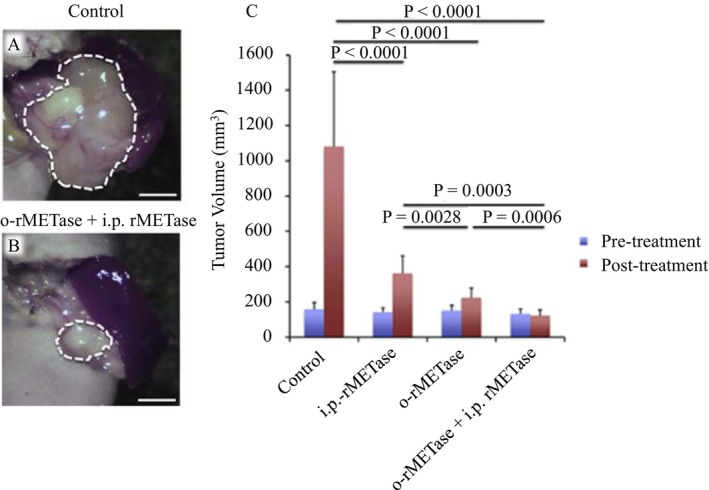
Oral rMETase is superior to injectable rMETase and overcomes acquired gemcitabine resistance in a pancreatic cancer PDOX model. A: Photograph of a tumor from a representative untreated control. B: Photograph of a tumor treated with the combination of o‐rMETase and ip‐rMETase in the pancreatic cancer PDOX. Tumors were resected on day 15. Scale bar: 5 mm. C: Quantitative efficacy of ip‐rMETase, o‐rMETase and their combination on the pancreatic cancer PDOX. Bar graphs show tumor volume at pre‐ and post‐treatment. Error bars:±SD. Reproduced from Kawaguchi et al. [60].

In PDOX models of highly recalcitrant cancers—including pancreatic cancer, melanoma, osteosarcoma, and sarcoma—rMETase showed impressive tumor suppression, particularly in combination with chemotherapeutics or *S. typhimurium* A1‐R [56, 57, 58, 59]. Of particular interest was the finding that oral administration of rMETase was unexpectedly effective, opening a promising route for clinical translation (Figure 6) [53, 54, 60, 61, 62, 63, 64, 65, 66, 67, 68, 69]. That a protein, in this case rMETase, could survive and be active in the gut was discovered by Japanese scientists working with Chinese scientists at AntiCancer Inc., an example of original “out‐of‐the‐box” thinking and international close collaboration and friendship [60]. We collaborated with PET imaging experts in Japan to visualize methionine uptake using 11C‐methionine PET, confirming the ‘Hoffman effect’ clinically [70]. Interestingly, rMETase lowered both plasma and intra‐tumoral methionine levels, synchronizing tumor cells into a more treatable phase of the cell cycle. Oral rMETase formulations now under development allow outpatient administration. Our trainees contributed to enzymatic optimization, pharmacokinetics, and co‐therapy designs, which paved the way for first‐in‐human studies [53, 54, 63, 64, 65, 66, 67, 68, 69, 71].

Japanese surgeon‐scientists trained with us contributed significantly to these studies. They refined rMETase production, validated its efficacy in diverse tumor types, and introduced this strategy into translational pipelines in Japan. Methionine restriction as a therapeutic axis represents one of the most innovative contributions of our group and continues to inspire new approaches to metabolic targeting in oncology.

## 7 Discussion and Future Outlook

Over the course of three decades, Japanese surgical trainees undertaking research in our laboratory in San Diego faced multiple challenges, particularly when transitioning from clinical environments into molecular biology‐based research. Many had limited prior experience with bench science and were unfamiliar with hypothesis‐driven investigation. To address this, we fostered a supportive, hands‐on learning environment that emphasized collaborative learning and personalized mentorship. Trainees gradually acquired experimental skills and deepened their understanding of cancer biology through immersive engagement with live‐imaging techniques, cancer models, and therapeutic interventions.

Upon returning to Japan, these surgeons were equipped not only with technical competencies but also with critical thinking skills and a mindset oriented toward scientific curiosity and innovation. Their experiences abroad broadened their clinical perspectives, encouraged interdisciplinary collaboration, and often inspired them to establish their own research initiatives within Japanese institutions. These returning surgeon‐scientists have played pivotal roles in bridging clinical and research domains, advancing both therapeutic development and surgical training methodologies.

The mindset of the “academic surgeon,” cultivated through international training, has contributed to professional development and research capacity. It has led to innovations in surgical techniques, fostered stronger connections between clinical practice and basic research, and inspired a new generation of surgical educators. As research institutions in Japan seek to rebuild their global standing, surgeon‐scientists with international experience represent a crucial human resource.

Nonetheless, systemic and cultural barriers persist, including limited funding opportunities, rigid training structures, and the undervaluing of academic output in clinical settings. To support the next generation, we advocate for increased institutional support for research sabbaticals, the establishment of integrated MD–PhD tracks, and greater recognition of academic contributions within surgical departments.

We encourage young Japanese surgeons to consider research not as a detour, but as an essential dimension of their professional growth. Mentorship, curiosity, and cross‐cultural engagement can serve as catalysts for both personal development and systemic change. The 30‐year story we share in this manuscript is not only one of scientific achievement, but of human connection and the sustained belief that surgeons can and should be scientists.

Despite the significant strengths of the methodologies described in this review, each approach has inherent limitations. For example, although PDOX models faithfully recapitulate the tumor microenvironment and metastatic patterns, they may gradually lose intratumoral heterogeneity and genetic fidelity over serial passages compared with the original patient tumor [72]. Such alterations can influence tumor behavior and drug response, underscoring the importance of periodic histopathological and molecular comparisons with the original tumor to ensure model validity [73]. FUCCI cell‐cycle imaging, while enabling real‐time visualization of tumor cell dynamics, is still in the early stages of clinical evaluation and remains confined to specific research settings. The A1‐R system is more effective in immunodeficient hosts for tumor targeting, which may not fully replicate the human immune context. It is promising that A1‐R is further developed in syngeneic immunocompetent mouse models as a bridge to the clinic. rMETase has shown promise in preclinical models; however, its long‐term effects on host metabolism and immunity are not yet fully understood. Continued refinement of these methodologies, along with integration of complementary model systems [74], will be valuable to further enhance their translational impact [75].

Looking ahead, strengthening international researcher exchange—particularly between Japan and the United States—will be critical for sustaining innovation in surgical oncology. Our three‐decade experience demonstrates that immersive overseas training has contributed to the professional development of Japanese surgeons. As Japan faces declining performance in international research indicators [76, 77], fostering structured bilateral fellowships, joint funding mechanisms, and virtual collaboration platforms will be essential. Encouraging returnees to serve as hubs that connect their home institutions with global networks may further amplify impact. Such measures can help ensure that Japanese surgeon‐scientists continue to contribute meaningfully to global research, while simultaneously revitalizing Japan's own academic environment.

## 8 Conclusion

Over the past three decades, our mentorship has helped shape a generation of Japanese surgeon‐scientists who now serve as principal investigators, professors, and department leaders. Many continue the cycle of mentorship, building a multigenerational, international network rooted in shared values of surgical excellence, scientific innovation, global collaboration, and most importantly, permanent friendship. What began as a small exchange between our San Diego lab and a few Japanese trainees has grown into a lasting academic movement. More than just technical training, our approach emphasized a philosophy—that science should be visual, dynamic, especially original, and deeply human. The seeds planted through this partnership continue to grow in research labs and operating rooms across Japan and beyond.

## Author Contributions

**Robert M. Hoffman:** conceptualization, writing – original draft, writing – review and editing, supervision. **Kentaro Miyake:** conceptualization, writing – original draft, writing – review and editing, data curation. **Itaru Endo:** writing – review and editing, supervision, conceptualization.

## Conflicts of Interest

Itaru Endo is an editorial board member of Annals of Gastroenterological Surgery.
